# Bacterial and fungal bioburden reduction on material surfaces using various sterilization techniques suitable for spacecraft decontamination

**DOI:** 10.3389/fmicb.2023.1253436

**Published:** 2023-12-13

**Authors:** Shunta Kimura, Shu Ishikawa, Nobuya Hayashi, Kazuhisa Fujita, Yuko Inatomi, Shino Suzuki

**Affiliations:** ^1^Institute of Space and Astronautical Science, Japan Aerospace Exploration Agency, Sagamihara, Japan; ^2^Space Exploration Innovation Hub Center, Japan Aerospace Exploration Agency, Sagamihara, Japan; ^3^Graduate Institute for Advanced Studies, SOKENDAI, Sagamihara, Japan; ^4^Engineering Division, Kajima Corporation, Tokyo, Japan; ^5^Interdisciplinary Graduate School of Engineering Sciences, Kyushu University, Fukuoka, Japan; ^6^Safety and Mission Assurance Department, Japan Aerospace Exploration Agency, Tsukuba, Japan; ^7^Geobiology and Astrobiology Laboratory, RIKEN Cluster for Pioneering Research, Wako, Saitama, Japan

**Keywords:** sterilization, bioburden reduction, planetary protection, forward contamination, spacecraft, microbial contamination

## Abstract

Planetary protection is a guiding principle aiming to prevent microbial contamination of the solar system by spacecraft (forward contamination) and extraterrestrial contamination of the Earth (backward contamination). Bioburden reduction on spacecraft, including cruise and landing systems, is required to prevent microbial contamination from Earth during space exploration missions. Several sterilization methods are available; however, selecting appropriate methods is essential to eliminate a broad spectrum of microorganisms without damaging spacecraft components during manufacturing and assembly. Here, we compared the effects of different bioburden reduction techniques, including dry heat, UV light, isopropyl alcohol (IPA), hydrogen peroxide (H_2_O_2_), vaporized hydrogen peroxide (VHP), and oxygen and argon plasma on microorganisms with different resistance capacities. These microorganisms included *Bacillus atrophaeus* spores and *Aspergillus niger* spores, *Deinococcus radiodurans*, and *Brevundimonas diminuta*, all important microorganisms for considering planetary protection. *Bacillus atrophaeus* spores showed the highest resistance to dry heat but could be reliably sterilized (i.e., under detection limit) through extended time or increased temperature. *Aspergillus niger* spores and *D. radiodurans* were highly resistant to UV light. Seventy percent of IPA and 7.5% of H_2_O_2_ treatments effectively sterilized *D. radiodurans* and *B. diminuta* but showed no immediate bactericidal effect against *B. atrophaeus* spores. IPA immediately sterilized *A. niger* spores, but H_2_O_2_ did not. During VHP treatment under reduced pressure, viable *B. atrophaeus* spores and *A. niger* spores were quickly reduced by approximately two log orders. Oxygen plasma sterilized *D. radiodurans* but did not eliminate *B. atrophaeus* spores. In contrast, argon plasma sterilized *B. atrophaeus* but not *D. radiodurans*. Therefore, dry heat could be used for heat-resistant component bioburden reduction, and VHP or plasma for non-heat-resistant components in bulk bioburden reduction. Furthermore, IPA, H_2_O_2_, or UV could be used for additional surface bioburden reduction during assembly and testing. The systemic comparison of sterilization efficiencies under identical experimental conditions in this study provides basic criteria for determining which sterilization techniques should be selected during bioburden reduction for forward planetary protection.

## 1 Introduction

Space exploration missions are promoted to comply with the Planetary Protection Policy and Requirements established by the Committee on Space Research (COSPAR) to ensure that future scientific investigations related to the chemical evolution of the solar-system bodies and the origin and distribution of life are not compromised (DeVincenzi et al., 1998; Nicholson et al., 2009; Kminek et al., 2019; COSPAR, 2021; Coustenis et al., 2023). The microbial contamination (bioburden) of flight systems, including landers or rovers, must be below the acceptable limit, especially for missions to Mars, Europa, Enceladus, and other solar-system bodies where the existence of extant life cannot be excluded (National Research Council, 2006; Grotzinger et al., 2012; European Cooperation for Space Standardization, 2019; Kminek et al., 2019; Rettberg et al., 2019; NASA, 2021, 2022).

Several sterilization methods are available for bioburden reduction, each with different mechanisms of effect, advantages, and disadvantages. Thus, a comparative study on sterilization methods against different microorganisms is essential to selecting appropriate techniques for bioburden reduction. Dry heat has been the standard method for sterilizing spacecraft since the Viking lander (Barengoltz and Stabekis, 1983; Grotzinger et al., 2012; European Cooperation for Space Standardization, 2013a; Shirey et al., 2017). However, this method is not applicable to non-heat-resistant materials such as electronics (European Cooperation for Space Standardization, 2013a): Dry heat also negatively affects metals, such as accelerated aging and possible softening (European Cooperation for Space Standardization, 2008a). UV irradiation is also widely used as a disinfection method. However, UV light can cause component surface degradation and cannot reach shaded areas. Alcohol disinfection is commonly used for bioburden reduction during spacecraft assembly, such as for the Mars Science Laboratory or Mars 2020 (Grotzinger et al., 2012; Farley et al., 2020); however, alcohol wets the object and only disinfects the surfaces. Isopropyl alcohol (IPA) is considered to be preferable over ethanol because some bacteria may use ethanol as a carbon source (Mogul et al., 2018). Treatment with aqueous solution and vapor of hydrogen peroxide (H_2_O_2_) is a non-residual method because it degrades into water and oxygen (Rohatgi et al., 2002; Kempf et al., 2005; Chung et al., 2006, 2008; European Cooperation for Space Standardization, 2013b; Rummel and Pugel, 2019). However, this method requires a high chemical concentration and may alter the material, including surface oxidation, strength loss, or volume change (European Cooperation for Space Standardization, 2008a). Plasma sterilization has the advantages of no residual chemicals and minimal adverse effects on components (Moisan et al., 2001, 2002; Hayashi et al., 2018; Choudhury et al., 2023); however, quantitative knowledge is still limited, and plasma sterilization has not been tested for spacecraft bioburden reduction.

We selected four microorganisms that are considered to be important in planetary protection based on previous studies (Patel et al., 2017; Craven et al., 2021; NASA, 2022): *Bacillus atrophaeus* is a gram-positive and spore forming bacterium, and its endogenous spores are commonly used for testing the resistance to dry heat, ethylene oxide, steam, and radiation (Setlow, 2006; Craven et al., 2021; NASA, 2022). In planetary protection, bioburden reduction aims to minimize the presence of entire microorganisms on spacecraft, with a specific focus on reducing the number of microbial spores that can survive a wet heat shock at 80°C for 15 min. This focus is considered crucial because spores have the capability to endure extremely harsh conditions for long periods (e.g., over 10,000 years), and in fact, for the mission of potentially habitable planet, the number of spores per spacecraft is documented in the Planetary Protection Policy (European Cooperation for Space Standardization, 2008b; Benardini et al., 2014a,b; Planetary Protection Independent Review Board, 2019). *Deinococcus radiodurans* is a gram-positive, non-spore forming bacterium known for their ability to survive exposure to strong radiation and desiccation in space (Anderson, 1956; Makarova et al., 2001; Cox and Battista, 2005; Kawaguchi et al., 2020). A gram-negative bacterium, *Brevundimonus diminuta* is a standard organism for validating the quality of sterilizing-grade membrane filters because its small cell size and monodispersed with a narrow size distribution (Sandle, 2013; Ryan and Pembroke, 2018; Roy, 2019). *Aspergillus niger* is a filamentous fungus whose spores are highly pigmented, resistant to UV-C radiation, and easily dispersed in the air (Schuster et al., 2002; Cortesão et al., 2020).

In addition to their different physiological properties, the four microorganisms have actually been detected in spacecraft related cleanrooms or labs. While a wide variety of microorganisms have been detected in spacecraft assembly and other related facilities by various methods, including incubation, DNA-based detection, and MALDI-TOF mass spectrometry, *Bacillus* spp. are often reported as the predominant species (Puleo et al., 1977; Venkateswaran et al., 2001; La Duc et al., 2004; Moissl-Eichinger et al., 2015; Koskinen et al., 2017; Hendrickson et al., 2021; Wood et al., 2021; Schubert et al., 2023). Of the bacteria detected on the six Mars spacecrafts, approximately 30% of those belonged to the genus *Bacillus*, 1–2% to the genus *Brevundimonas,* and 1–2% to the genus *Deinococcus* (Schwendner et al., 2020). Kminek et al. reported that the genera *Bacillus* and *Brevundimonas* accounted for 9 and 4% of the microorganisms in the cleanroom, respectively (Kminek et al., 2019). The genus *Deinococcus* was also often detected, and reported that they were in all NASA’s cleanrooms at the Jet Propulsion Laboratory, Kennedy Space Center, and Johnson Space Center (Moissl et al., 2007; Wood et al., 2021). Cultivable fungi were also detected in the cleanroom in spacecraft assembly facility of NASA (Hendrickson et al., 2017). Regberg et al. (2018) reported that between 83 and 97% of the microorganisms were fungi in the meteorite lab cleanrooms at NASA.

To select suitable sterilization techniques for bioburden reduction for forward planetary protection, we compared the effects of dry heat, UV light, IPA, H_2_O_2_, VHP, and oxygen and argon plasma on four different microorganisms in this study. A colony counting method, gold standard for sterilization tests, was used to evaluate living organisms after microbial reduction treatment (European Cooperation for Space Standardization, 2008b; Patel et al., 2017; Chen et al., 2023). *Bacillus atrophaeus* and *A. niger* were used in the spore state as represented the bacterial and fungal spores. *Deinococcus radiodurans* and *B. diminuta*, which do not form spores, were used as dried vegetative cells. Based on comprehensive and systematic information, we proposed the optimal bioburden reduction process of spacecraft.

## 2 Materials and methods

### 2.1 Microbial strains and culture conditions

A list of the microorganisms used in this study is shown in Supplementary Table S1. *Bacillus atrophaeus* NBRC 13721, *D. radiodurans* NBRC 15346, and *B. diminuta* NBRC 14213 were purchased from NITE Biological Resource Center (NBRC; Chiba, Japan). *Aspergillus niger* ATCC 16888 was purchased from Microbiologics (St. Cloud, MN, United States). *Deinococcus radiodurans* and *B. diminuta* were cultivated in NBRC No. 702 liquid medium [10 g Hipolypepton (390-02116, Fujifilm Wako, Japan), 2 g yeast extract, and 1 g MgSO_4_·7H_2_O per liter; pH 7.0] at 30°C, with shaking at 120 rpm (WB-205QMC, WakenBtech, Japan). *Bacillus atrophaeus* spores were harvested from the agar plates after 10 days of incubation at 37°C for spore formation [8 g nutrient broth, 4 g yeast extract, 0.0157 g MnCl_2_·4H_2_O, and 15 g agar/L] by gently scraping with sterilized saline solution (0.9% NaCl) using a cell spreader. The spores were washed thrice with saline solution, incubated at 80°C for 15 min, washed twice with ethanol, and rewashed with saline solution. *Aspergillus niger* spores were harvested after 10 days of incubation at 25°C from NBRC No. 5 agar plates [20 g malt extract, 20 g glucose, 1 g peptone, and 20 g agar/L; pH 6.0] by gently stroking with a droplet of 0.05% TritonX-100 solution using a Pasteur pipette. The spores were suspended in a sterilized saline solution (0.9% NaCl). Spore formation was determined using phase contrast microscopy (IX73, Olympus, Japan) and the spore content was approximately 90%.

### 2.2 Preparing inoculated coupons for bioburden reduction experiments

**Figure 1:**
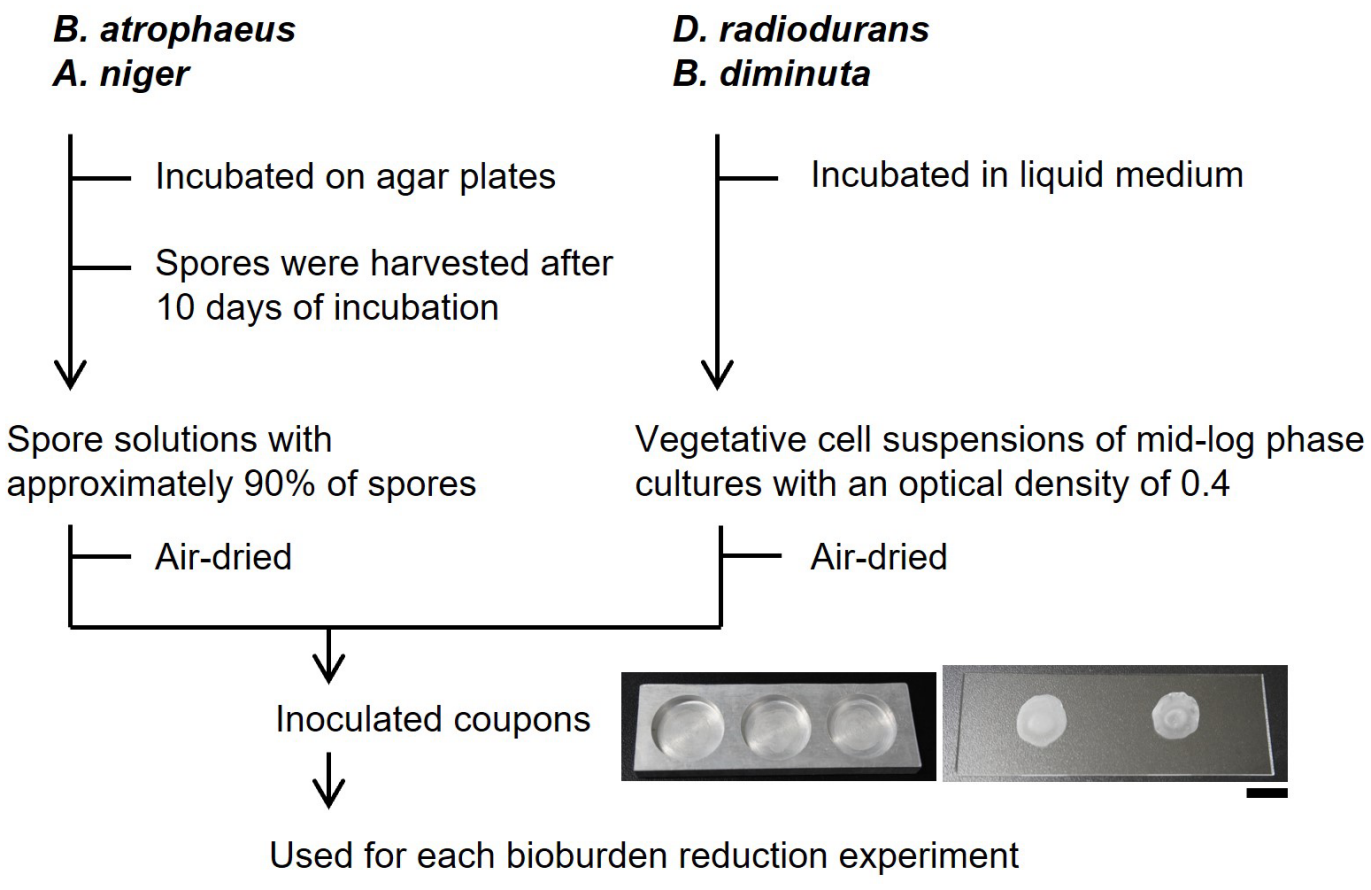
Schematics of bioburden reduction experiments. The scale bar shows 1 cm.

The vegetative cell suspensions (10 or 50 μL) of mid-log phase cultures with an optical density of 0.4 (CO7500, Funakoshi, Japan) of *D. radiodurans* and *B. diminuta* and the 10 μL of *B. atrophaeus* spore solutions and 50 μL of *A. niger* spore solutions were dropped onto glass slides and aluminum plates with holes (φ18 mm and a depth of 2.8 mm) and air-dried for over 1 h in biological safety cabinets (AC2-4 N7, Esco, Singapore). The glass slide coupons were used for bioburden reduction experiments using dry heat, UV, vaporized hydrogen peroxide (VHP), and oxygen and argon plasma (Figure 1). The aluminum plate coupons were used for the bioburden reduction experiments using 70% IPA and 7.5% H_2_O_2_. Approximately 6–7 log orders of bacterial cells (including spores) and 4–5 log orders of fungal spores per 1 cm in diameter were dried on each plate. Coupons used as controls were prepared at the same time as the samples, stored in desiccators at a relative humidity of ≤30% and a temperature of 22.8°C, and incubated for the same time as the samples. Drying, microbial reduction treatment, collection, and evaluation of live cells were performed within 30 days for the plasma sterilization test and 7 days for the other tests. After drying, survival during inoculated coupons storage was examined by placing them in the desiccators. The thicknesses of surface-attached microbial cells or spores were measured by calculating the average height over 100 μm horizontally (*n* ≥ 8) using a laser microscope (VK-X 100, Keyence, Japan).

### 2.3 Dry heat treatment

Dry heat treatment was performed in a dry oven (MOV-212S, Sanyo, Japan). The treatment time was measured from when the samples were placed in the preheated oven (100, 112, 120, 140, 160, and 180°C) until removal. Inoculated coupons were covered in aluminum foil throughout the treatment. After the treatment, inoculated coupons were removed from the oven and cooled at 22.8°C. The surface temperatures on the inoculated coupons were measured using a data logger (GL240, Graph Tech, Japan).

### 2.4 UV irradiation

UV irradiation was performed using a UV lamp with a 253.7 nm wavelength (GL 15, Toshiba, Japan) mounted on a clean bench (MCV-131BNS, Sanyo, Japan). The UV intensity was measured using a data logger (YK-37UVSD, Mothertool, Japan), and the UV dose was adjusted to 1.26 J/cm^2^/h. The relative humidity was <60%. Throughout the treatment, inoculated coupons were not exposed to intense light sources other than the UV lamp to prevent the photoactivation of the cellular DNA repair systems mediated by photolyase.

### 2.5 Isopropyl alcohol or H_2_O_2_ treatment

Alcohol and H_2_O_2_ treatment were performed by adding 250 μL of 70% IPA (2-propanol; 32435-2B, Kanto Chemical, Japan) and 7.5% H_2_O_2_ (20779-65, Nacalai Tesque, Japan) to the samples, respectively. The concentrations followed the methods of the Mars Organic Molecule Analyzer for bioburden-controlled clean rooms (Lalime and Berlin, 2016). After the treatments, each sample was diluted with sterilized saline solution (0.9% NaCl) to >200-fold for IPA and > 20,000-fold for H_2_O_2_ to avoid cell death by residual IPA or H_2_O_2_. We have confirmed that microbicidal activities did not continue under each diluted condition.

### 2.6 Vaporized hydrogen peroxide treatment

Vaporized hydrogen peroxide treatments were performed using a vacuum chamber (diameter: 210 mm; length: 495 mm; volume: 17.1 L). The chamber was evacuated, and 35% of liquid H_2_O_2_ was supplied from the top of the reactor through continuous evacuation using a vacuum pump and was gasified under a pressure of 400 ± 20 Pa. The temperature inside the vacuum chamber was maintained at 22.8°C. Hence, the relative humidity and concentration of VHP in the treatment were calculated as <11.6% RH and 1.07 ± 0.06 mg/L, respectively. The total moles of mixed gas comprising H_2_O and H_2_O_2_ derived from 35% H_2_O_2_ (by weight) were calculated as 2.44 mmol using the ideal gas law as the pressure in the evacuated chamber was 50 Pa without 35% H_2_O_2_. Subsequently, the moles of VHP molecules in the chamber were determined as 0.54 mmol (18.4 mg) according to Dalton’s law. The concentration-time (CT) values of VHP treatment were calculated as the product of VHP concentration and exposure time.

### 2.7 Plasma treatment

Plasma treatments were performed using a capacitively coupled radio-frequency discharge reactor (Hayashi et al., 2006, 2018). The discharge electrode was a serpentine folding-shaped stainless-steel wire (diameter: 4 mm; total length: 1,800 mm) placed approximately 20 mm beneath the upper wall of the reactor. Pure oxygen or argon gas was supplied from the top of the reactor with a pressure of 60 Pa. A radio-frequency voltage of 13.56 MHz was applied to the discharge electrode. The discharge power was maintained at 80 W. The temperature inside the vacuum chamber was maintained at <65°C. The samples were placed 90 mm below the top of the discharge electrode.

### 2.8 Colony count

After each microbicidal treatment, the microbial cells attached to inoculated coupons were collected by pipetting in triplicate using a total of 100 μL NBRC No. 702 medium. After dilution with NBRC No. 702 media, *B. atrophaeus*, *B. diminuta*, and *D. radiodurans* cells were inoculated on NBRC No. 802 agar plates [pH 7.0, composition: 10 g hipolypepton (390-02116, Fujifilm Wako, Japan), 2 g yeast extract, and 1 g MgSO_4_·7H_2_O, and 15 g agar/L]. *Aspergillus niger* spores were inoculated on potato dextrose agar plates (Solabia, France). The agar plates were incubated at 30°C for bacteria and 25°C for fungi in an incubator (IC602, Yamato, Japan): 24–48 h for *B. atrophaeus* cells and 72–96 h for the other microorganisms. The resultant colonies were counted (Scan 300, Interscience, France) to calculate the survival rate. All experiments were performed simultaneously on two dry microbial pellets, and the values were averaged. All experiments were repeated at least once for confirmation of repeatability. Cells collection was confirmed by colony counting and the repeatability was approximately 80%.

### 2.9 Thermal cell death kinetic models

During dry or moist heat treatment, the thermal death of microbial cells generally follows the first-order of kinetics (Pflug et al., 2001) as described by Equation (1)


(1) $$-\frac{dN}{dt}=k_{T}N$$

where *N* and *t* are the viable cell number and time (min or h), respectively, and *k_T_* is the death rate constant (min^−1^ or h^−1^) at a constant heating temperature *T* (°C). When taking the integral of both sides of Equation (1), Equation (2) is obtained using *N*_0_ as the initial viable cell number.


(2) $$\log\frac{N}{N_{0}}=\left(-\frac{k_{T}}{\ln10}\right)t$$

The *D*-value (*D_T_*, min or h) is the time required to reduce a microbial population by 10-fold at heating temperature *T* and is calculated from the slope of the line obtained from the logarithmic plot of survival rate, log (*N*/*N*_0_) against *t*. Therefore, Equation (3) can be described by Stoforos (2015) as:


(3) $$D_{T}=\frac{\ln10}{k_{T}}$$

The *z*-value (°C) is defined as the change in temperature that will increase the *D*-value by a factor of 10 and is also calculated from the slope of the line obtained from the plot of log *D_T_* against *T*.

### 2.10 *F*-value

The *F*-value (min or h) is the heating time equivalent at any heating temperature *T* for a specified *z*-value and reference temperature *T*_R_ (Moldenhauer, 2013). Generally, the *F*-value is obtained through Equation (4),


(4) $$F_{T_{R}}=\int L_{T}dt=\int \frac{k_{T}}{k_{T_{R}}}dt=\int 10^{\left(T-T_{R}\right)/z}dt$$

where *L_T_* is the lethal rate defined as the constant death rate at temperature *T* divided by that of reference temperature *T*_R_. When *T* is a constant value, Equation (5) is obtained.


(5) $$F_{T_{R}}=10^{\left(T-T_{R}\right)/z}t=10^{\left(T-T_{R}\right)/z}F_{T}$$

During heating and cooling periods, thermal processes were performed under time-varying temperatures *T*(*t*), and the *F*-value is obtained by Equation (6), where *F*_P_ is the *F*-value of the process, which also approximates Equation (7).


(6) $$F_{P}=\int L_{T\left(t\right)}dt=\int \frac{k_{T\left(t\right)}}{k_{T_{R}}}dt=\int 10^{\left(T\left(t\right)-T_{R}\right)/z}dt$$

(7) $$F_{P}≅\overset{n}{\underset{n=1}{\sum }}L_{\bar{T}\left(\Delta t_{n}\right)}\Delta t_{n}=\overset{n}{\underset{n=1}{\sum }}10^{\left(\bar{T}\left(\Delta t_{n}\right)-T_{R}\right)/z}\Delta t_{n}$$

In Equation (7), $\bar{T}\left(\Delta t_{n}\right)$ is the average temperature of the process at the time interval $\Delta t_{n}$ . Additionally, using Equation (5), the *F*_P_-values between two thermal processes with different processing temperatures *T*_1_ and *T*_2_ can be described as Equation (8).


(8) $$F_{P,T1}=10^{\left(T2-T1\right)/z}F_{P,T2}$$

Generally, when the *F*-value of the dry heat sterilization is calculated, a *z*-value of 20°C is used as that temperature is indicated as the *z*-value of the bacterial spores (Gould, 2004).

### 2.11 Determining the accuracy of the processing time length in each dry heat treatment using the *F*_P_-value

The *F*_P_-value of each dry heat treatment was obtained using Equation (7). The total processing time, *t,* was first divided into *n* pieces of segments, each with an interval of 20 s. Subsequently, *L*-values at the average temperature of each time segment were calculated and added (Supplementary Table S2; Supplementary Figures S1, S2). In this calculation, the processing temperature of each dry heat treatment was considered as each reference temperature *T*_R_, and a *z*-value of 20°C was used. When the processing time value, *t,* was markedly shorter than the obtained *F*_P_, survival rate data at that time were not used to estimate the *D*-values.

## 3 Results

### 3.1 Properties of simulated bioburden attached to test coupon surfaces

No substantial changes in the colony-forming units of dried *B. atrophaeus* and *D. radiodurans* cells were observed after a storage period of over 30 days (Supplementary Figure S3). The change in dried *A. niger* after 30 days of storage was less than one log reduction. In contrast, dried *B. diminuta* cells showed a one log reduction after 7 days of storage and an approximately three log reduction after 1 month. Supplementary Table S3 shows the thickness of dried cells (approximately 1 cm in diameter) on the glass slide used in the bioburden reduction tests. The dried *D. radiodurans* and *B. diminuta* cells tended to become thicker than those of *B. atrophaeus* and *A. niger*. The cells thicknesses of *B. atrophaeus* and *A. niger* locally exceeded 10 μm due to clumped cells.

### 3.2 Dry heat treatment

**Figure 2:**
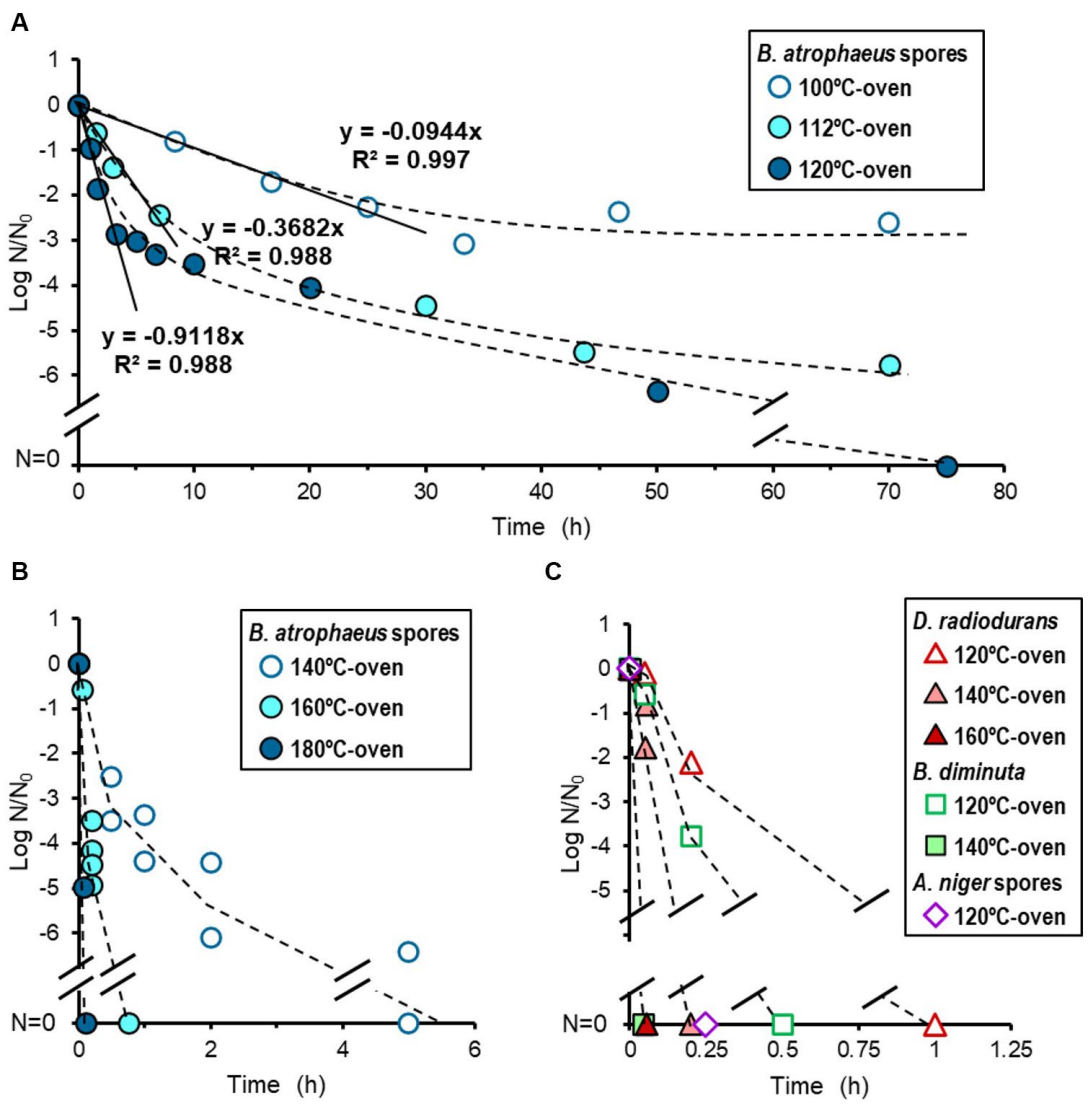
Microbial reduction after dry heat treatment. Reduction curves of *Bacillus atrophaeus* spores heated using a 100-, 112-, and 120°C-oven **(A)** and a 140-, 160-, and 180°C-oven **(B)**. Reduction curves of *Deinococcus radiodurans*, *Brevundimonas diminuta*, and *Aspergillus niger* spores **(C)**.

While each dry heat treatment was set at 100, 112, 120, 140, 160, and 180°C, the actual temperatures were 100.8 ± 0.2, 113.4 ± 0.2, 122.0 ± 0.3, 141.9 ± 0.5, 161.3 ± 0.6, and 181.8 ± 0.4°C, respectively. The results for dry heat treatment set at 100-, 112-, and 120°C for *B. atrophaeus* are shown in Figure 2A. The reduction curves obtained from these temperatures were linear for two or three log orders. Subsequent microbial reduction showed tailing. The *D*-values for 100-, 112-, and 120°C-heat treatments were calculated as 604, 163, and 65.8 min, respectively. The dry heat treatment of *B. atrophaeus* exposed to 140-, 160-, and 180°C are shown in Figure 2B. Over six log orders of spores could be inactivated at 140°C for 5 h, 160°C for 45 min, and 180°C for 6 min. However, the *D*-values could not be determined for these treatments because the survival rate values dropped before the temperature of the inoculated coupons reached a steady state (Supplementary Table S2; Supplementary Figures S1, S2). *Deinococcus radiodurans*, *B. diminuta*, and *A. niger* dry heat treatment results are shown in Figure 2C. The three microorganisms were sterilized (i.e., under detection limit) more rapidly than *B. atrophaeus* (Table 1). All strains tested in this study were sterilized using a dry heat set at 120°C and above.

**Table 1:** Treatment time required to sterilize the surficial bioburden using dry heat, UV irradiation, and treatment with antimicrobial agents, including vaporized hydrogen peroxide (VHP) and oxygen- and argon plasma.

Treatment		Microorganism			
		*Bacillus atrophaeus* NBRC13721 (spores)	*Deinococcus radiodurans* NBRC15346	*Brevundimonas diminuta* NBRC14213	*Aspergillus niger* ATCC16888 (spores)
Dry heat^†^	Treated in an oven maintained at 120°C	75 h (7.3)^*^	<1 h (5.8)	<0.5 h (5.7)	<0.5 h (4.2)
	Treated in an oven maintained at 140°C	*ca.* 6 h (7.7)	<0.5 h (5.9)	<0.5 h (6.4)	Not tested
UV irradiation	Exposure to UV light (253.7 nm) at a dose of 1.26 J/cm^2^/h	*ca.* 4 h (7.2)	30 h (5.9)	*ca.* 4 h (5.6)	Not achieved at 30 h (3.2–3.6)
Antimicrobial agents	70% isopropyl alcohol (IPA), treatment for 10 min	Not achieved at 10 min (<1.0)	<5 min (6.5)	<5 min (6.4)	<5 min (4.5)
	7.5% hydrogen peroxide, treated for 10 min	Not achieved at 10 min (<1.0)^§^	<5 min (>4.6)	<5 min (>4.3)	Not achieved at 10 min (<1.0)^§^
Vaporized hydrogen peroxide (VHP)^‡^	CT values of the treatment were increased by 64.2 mg∙min/L per hour (400 Pa, 22.8°C, <11.6%RH)	Not achieved at 9 h (2.0–2.9)	<0.5 h (6.4)	<0.5 h (6.4)	Not achieved at 9 h (3.8)
Plasma	Oxygen-plasma	Not achieved at 8 h (4.1–4.8)	*ca.* 8 h (6.3)	Tested, but data are insufficient	<2 h (4.3)
	Argon-plasma	2–4 h (7.2)	Not achieved at 8 h (1.4–1.6)	Tested, but data are insufficient	4 h (4.4)

^*^Numbers in parentheses indicates log reduction values of bioburden. ^†^Processing temperature at the coupon surfaces were 122.0 ± 0.3 and 141.9 ± 0.5°C in a 120- and 140°C-oven, respectively. ^‡^VHP was supplied and discharged continuously at 0.932 mg/L/min via vacuuming. ^§^When the samples were treated for over 60 and 30 min, 6 log and 4 log reductions were observed in *Bacillus atrophaeus* and *Aspergillus niger* spores, respectively.

### 3.3 UV irradiation

**Figure 3:**
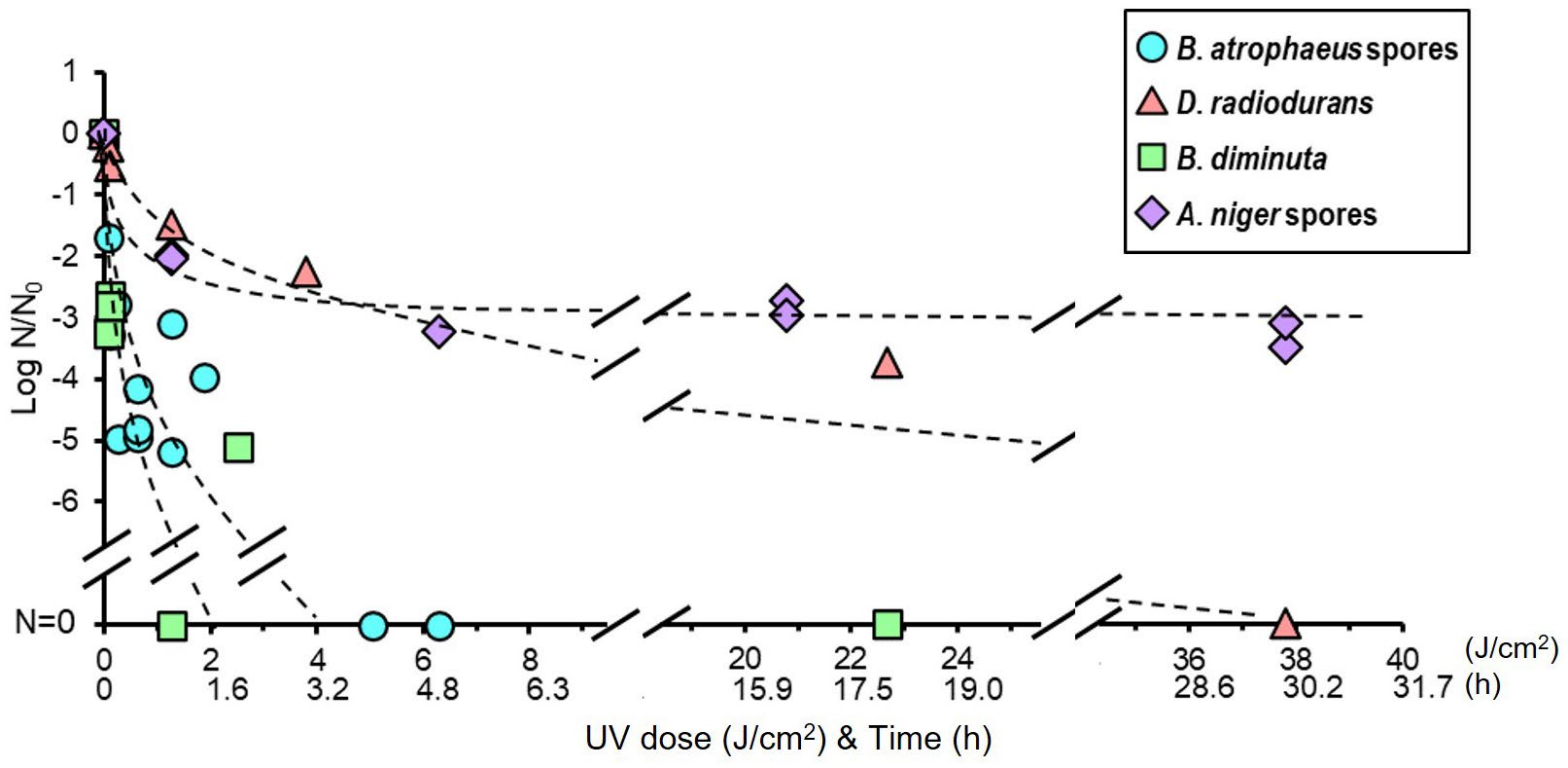
Microbial reduction after UV light irradiation of dry *Bacillus atrophaeus* spores, *Deinococcus radiodurans*, *Brevundimonas diminuta*, and *Aspergillus niger* spores.

The reduction curve of UV treatment is shown in Figure 3. The irradiation intensity measured by the UV lamp at 32.7 cm was 0.35 ± 0.1 mW/cm^2^, equal to 1 J/cm^2^ for 47.62 min of irradiation. An approximately five log reduction was observed in *B. atrophaeus* and *B. diminuta* after a UV irradiation of 2.5 J/cm^2^ (2 h irradiation). In contrast, UV light irradiation resistance was observed for *D. radiodurans* and *A. niger*. For both strains, a tailing of the survival curve was observed after the 1.5–2 log reduction in viable cells at a UV dose of 1.26 J/cm^2^ (1 h irradiation). *Deinococcus radiodurans* required a UV dose of 37.8 J/cm^2^ (irradiate for 30 h) for sterilization, whereas *A. niger* remained at a 2–3 log reduction in viable cells after the same treatment. These results suggested that the UV sterilization was effective for *B. diminuta* and *B. atrophaeus* but relatively ineffective for *D. radiodurans* and *A. niger* (Table 1), differing from the dry heat treatment results.

### 3.4 Treatment with alcohol and H_2_O_2_

**Figure 4:**
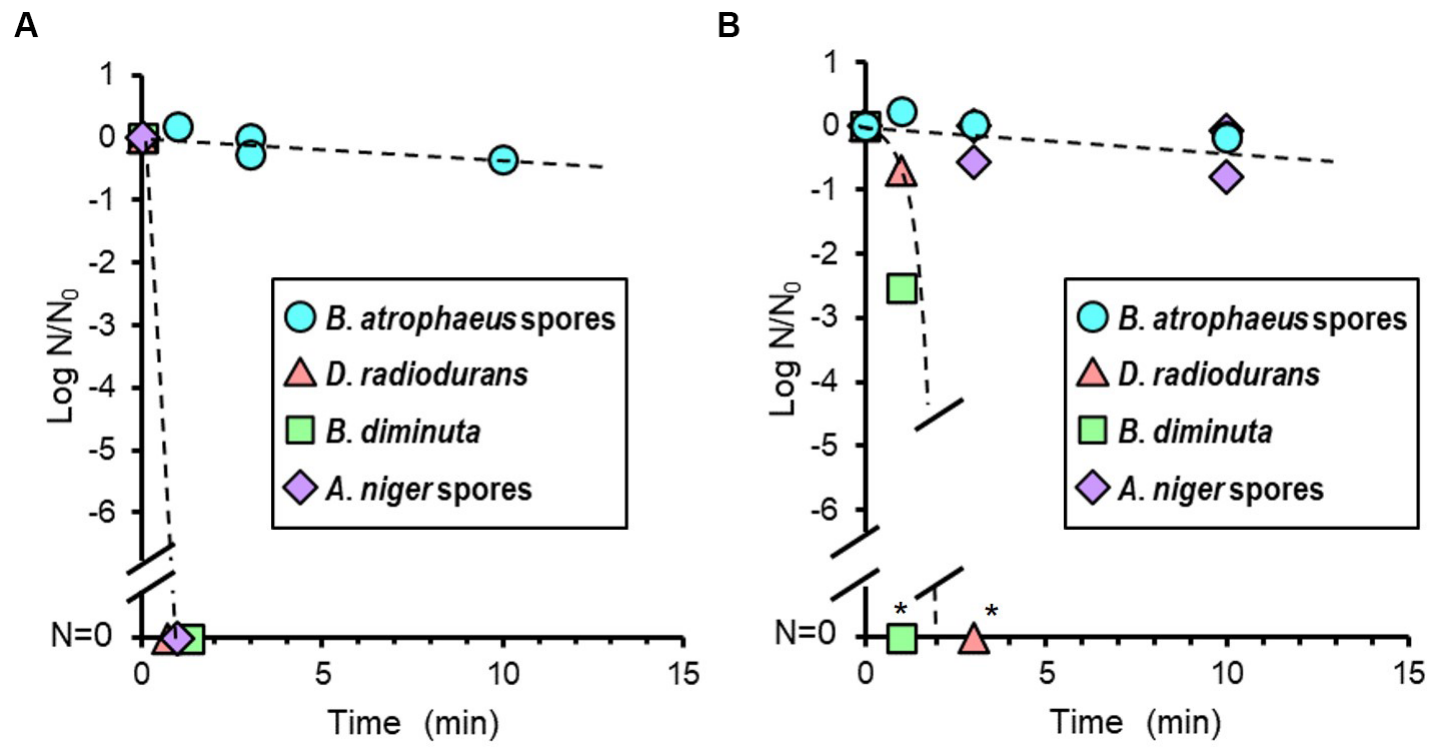
Microbial reduction after 70% of isopropyl alcohol (IPA; **A**) and 7.5% hydrogen peroxide (H_2_O_2_) treatments **(B)** of dry *Bacillus atrophaeus* spores, *Deinococcus radiodurans*, *Brevundimonas diminuta*, and *Aspergillus niger* spores. ^*^Due to the dilution associated with processing steps, the dynamic range is <5 log order.

The reduction curve after alcohol treatment is shown in Figure 4A. Seventy percent IPA solution was dropped onto the dried cells, and no suspension or wiping treatment was performed. *Brevundimonas diminuta*, *D. radiodurans*, and *A. niger* were sterilized quickly within a few minutes. However, no *B. atrophaeus* sporicidal activity was observed even when IPA infiltration was continued for 120 min.

The reduction value of viable cells after H_2_O_2_ treatment is shown in Figure 4B. H_2_O_2_ treatment was performed similarly to the IPA treatment. *Brevundimonas diminuta* and *D. radiodurans* were also sterilized quickly. In contrast, *B. atrophaeus* and *A. niger* microbicidal activities were scarcely observed after 10 min of treatment. Continuous H_2_O_2_ treatments inactivated more than four log orders of *A. niger* spores after 30 min and over six log orders of *B. atrophaeus* spores in almost all tests after 60 min (data not shown).

### 3.5 VHP treatment

Vaporized hydrogen peroxide treatment was performed under low pressure (400 ± 20 Pa) at 22.8°C with a relative humidity of ≤11.6%. VHP was continuously supplied and exhausted at 0.93 ± 0.06 mg/L/min throughout the treatment (Supplementary Figure S4). The VHP concentration was maintained at 1.07 ± 0.06 mg/L in the chamber. The CT value of the treatment increased by 64.2 mg·min/L every hour.

**Figure 5:**
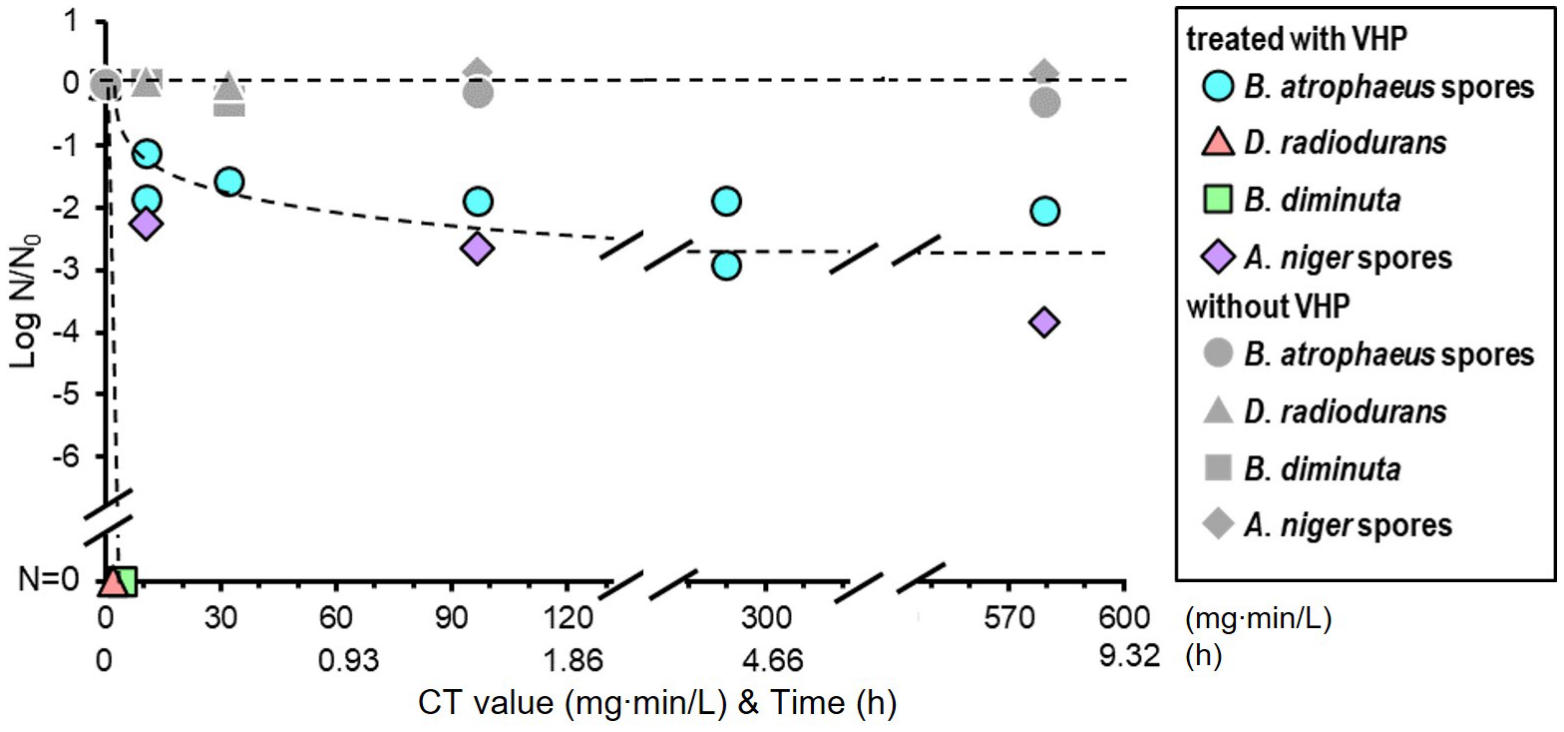
Microbial reduction after vaporized hydrogen peroxide (VHP) treatments of dry *Bacillus atrophaeus* spores, *Deinococcus radiodurans*, *Brevundimonas diminuta*, and *Aspergillus niger* spores.

The VHP treatment results are shown in Figure 5. *Brevundimonas diminuta* and *D. radiodurans* were immediately sterilized; however, *A. niger* and *B. atrophaeus* were inactivated by one to two log orders after 10 min of exposure, corresponding to the CT value of 10.7 mg∙min/L, and further effect was not observed. No substantial reduction was observed in vacuum-only treatment for all microorganisms in this study.

### 3.6 Oxygen- and argon plasma treatment

**Figure 6:**
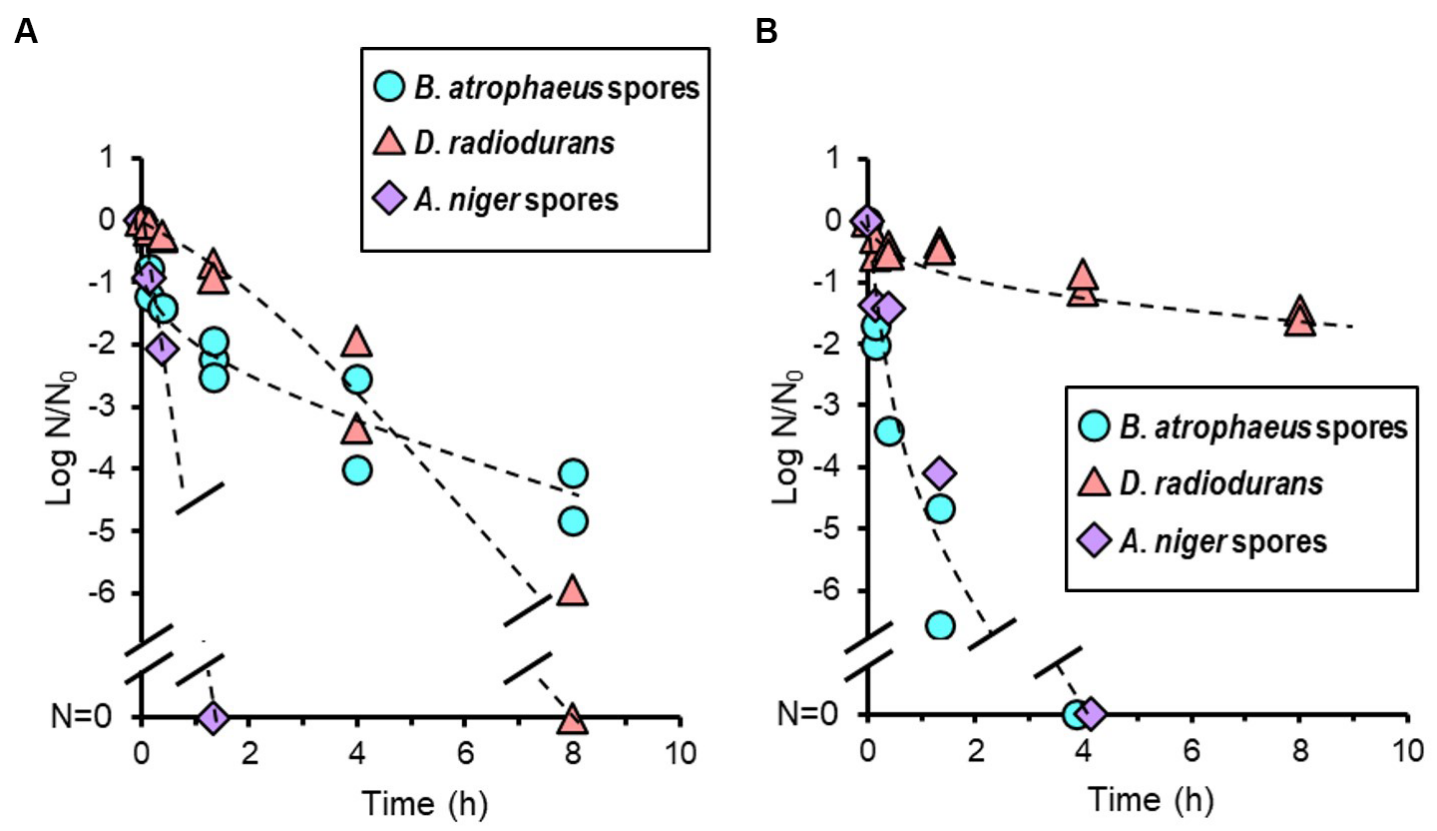
Microbial reduction after oxygen **(A)** and argon plasma treatments **(B)** of dry *Bacillus atrophaeus* spores, *Deinococcus radiodurans*, and *Aspergillus niger* spores.

Temperatures in the vacuum chamber during oxygen- and argon plasma treatment were increased during the first 20 min and reached constant values of 64 and 59°C, respectively. The reduction curves of test strains after oxygen plasma treatment are shown in Figure 6A. *Aspergillus niger* was sterilized after 1.3 h of treatment. *Deinococcus radiodurans* showed a reduction curve with a shoulder and was sterilized after 8 h. *Bacillus atrophaeus* showed a reduction curve with a tailing, and approximately two and four log orders of reduction in survival spores were observed at 1.3 and 8 h, respectively. The reduction curve after argon plasma treatment is shown in Figure 6B. Approximately 1.5 log orders of *D. radiodurans* cells were killed after 8 h of treatment; however, sterilization was incomplete under these conditions. In contrast, *B. atrophaeus* and *A. niger* were sufficiently sterilized within 4 h. Plasma treatment can reduce *B. atrophaeus* spores by three or more log order; however, the survival of *D. radiodurans* must be investigated. *Brevundimonas diminuta* sterilization using oxygen- and argon plasma treatment has been confirmed with at least two log reductions (data not shown); however, sufficient initial cell numbers were not prepared because *B. diminuta* died during dry storage (Supplementary Figure S3).

## 4 Discussion

### 4.1 Relationships of sensitivity between microbial samples and bioburden reduction techniques

A spacecraft is composed of several essential components, including an engine, power subsystem, steering system, communications system, and scientific instruments. Typically, it operates in space for several years. Consequently, planetary protection engineers must carefully choose suitable sterilization methods to ensure to reduce microbial cells without causing any damage to the spacecraft components. This study is valuable for this purpose, as it meticulously compares representative sterilization methods and provides sterilization efficiencies in *D-* or *z-*values wherever feasible.

*Bacillus atrophaeus* had the highest resistance to dry heat, 70% IPA, 7.5% H_2_O_2_, and VHP compared to the other strains (Table 1). In contrast, *B. atrophaeus* did not show resistance to UV light irradiation and argon plasma. *Deinococcus radiodurans* showed higher resistance to UV light and argon plasma than *B. atrophaeus* but did not show resistance to other treatments, except for oxygen plasma treatment. *Aspergillus niger* was resistant to UV light, similar to *D. radiodurans*, and also showed resistance to 7.5% H_2_O_2_ and VHP, like *B. atrophaeus*. *Brevundimonas diminuta* did not show resistance to any bioburden reduction method used in this study. *Brevundimonas diminuta* are sterilized when treated under conditions that would inactivate *B. atrophaeus*, *D. radiodurans*, and *A. niger*.

### 4.2 Microbicidal mechanisms and properties of sterilization methods

Logarithmic microbial cell death is theoretically observed when cellular damage from the sterilization treatment is homogeneous within the microbial cell population, which is often seen when cells are in a liquid solution. Inoculated coupons used in this study had a mass of air-dried vegetative or spored cells attached to the surface of the material; thus, the cellular damage in the microbial population tended to be heterogeneous since the damage caused by sterilization can differ depending on the location of the cells.

Heat sterilization, either dry or wet, is known to be effective. Air-dried *D. radiodurans* and *B. diminuta* cells and *B. subtilis* spores are reported to be easily killed, presumably by protein denaturation and DNA damage (Setlow, 2006). The tailing portion of the reduction curve of *B. atrophaeus* treated with dry heat were observed (Figure 2A) It is possible that there is variation in heat sensitivity within the inoculated *B. atrophaeus* population, resulting in a decrease in more sensitive cells during the earlier stages of the bioburden reduction process. Compared to the dry heat treatment conducted by Kempf et al. (2008) and Schubert and Beaudet (2011) who used an oil bath or stainless-steel vessel cylinders, our dry heat treatment (oven heating) was closer situation to the dry heat chamber of spacecraft component for bioburden reduction. However, in this study, the velocity during the heat-up or cool-down processes was slower than that described by Kempf et al. (2008) and Schubert and Beaudet (2011). The coupon temperature (processing temperature) took approximately 20 min to reach a steady-state temperature condition at any oven temperature during dry heat treatment (Supplementary Figures S1, S2). The accuracy of the processing time was confirmed using the *F*_P_-value (Supplementary Table S2). The *D*-values were calculated when the heating time during the non-steady state (heating and cooling) could be ignored compared to the total processing time of dry heat treatments. The reduction curve for *B. atrophaeus* ATCC9372 (synonym for NBRC13721) spores obtained in this study agrees with that reported by Kempf et al. (2008) and Schubert and Beaudet (2011), with an estimated *z*-value of 22.0°C, similar to the *z*-value of general bacterial spores during dry heating (Supplementary Figure S5).

UV light is known to cause DNA damage, leading to microbial inactivation (Horneck et al., 1984, 2006, 2010; Horneck, 1993). *Deinococcus radiodurans* and *A. niger* are reported to have strong radioresistance due to their superior DNA repair mechanism (Cox and Battista, 2005; Cortesão et al., 2020). Our results also showed that the UV light resistance was higher in these species than the other microorganisms (Figure 3). The inoculated *A. niger* coupons used in our bioburden reduction experiments were approximately 4 μm thick (Supplementary Table S3) and > 10 μm thick locally. The tailing off in the reduction curve of the UV treatment may be caused by the fact that only the cells at the outermost layer of the multi-layered structure were killed by the UV light and the UV light could not reach cells located within the inner position of the layer (Kawaguchi et al., 2020; Cortesão et al., 2021; Schuerger and Headrick, 2023). The *B. atrophaeus* and *A. niger* were suspended in the saline solution and *D. radiodurans* and *B. diminuta* were suspended in media before dropped on the coupon, so the ingredients of the incubation medium might shield against UV treatment in the test of *D. radiodurans* and *B. diminuta* and the UV radiation to sample coupon without media can be more effective.

Treatment with three different chemical agents, IPA, H_2_O_2_, and VHP, also exhibited varying sterilization profiles among the four microorganisms. Alcohol is thought to be a nonspecific antimicrobial agent because of the diverse toxicity mechanisms (Ali et al., 2001) and treatment with H_2_O_2_, which is mediated by the hydroxyl radical (•OH) generated by the Fenton reaction with intracellular iron, also presumably kill any types of microorganisms (Repine et al., 1981; Clifford and Repine, 1982). However, only slight reduction was observed when *B. atrophaeus* were treated with 70% IPA for 120 min or 7.5% H_2_O_2_ for 10 min and when *A. niger* was treated with 7.5% H_2_O_2_ for 10 min (Figure 4). VHP treatment is more effective to the spores of *B. atrophaeus* and *A. niger* but they could not be completely sterilized with VHP as well in this study (Figure 5). All these indicated that chemical treatment may not be quite effective to the fungal and bacterial spores due to the lower envelope permeabilities of spored cells although the mechanism of action of VHP may differ from that of liquid H_2_O_2_ (Linley et al., 2012). Meanwhile, *D. radiodurans* and *B. diminuta* were quickly sterilized using VHP (Figure 5), as with IPA and liquid H_2_O_2_.

The mechanism of oxygen-plasma sterilization is considered an etching effect of the cell surface by oxygen plasma and cell membrane disruption by hydroxyl radicals and atomic oxygen. The heat generated as a side effect may also have bioburden reduction effects. The mechanism of argon plasma bioburden reduction is also an etching of the cell surface and the UV light emitted from de-excitation particles in the plasma (Moisan et al., 2001, 2002). Interestingly, oxygen and argon plasma showed different sterilization spectra from any other sterilization techniques used in this study (Figure 6). Oxygen and argon plasma inactivated *A. niger*. Argon plasma sterilized *B. atrophaeus*, whereas *D. radiodurans* was not sterilized, differing from the oxygen plasma results but similar to its response to UV light. However, although *A. niger* was UV light-resistant, it was quickly sterilized; therefore, other mechanisms could contribute to *D. radiodurans* being resistant.

### 4.3 Bioburden reduction strategies for spacecraft

Decontamination processes of spacecraft components can be divided into initial bioburden reduction prior to delivery to assembly facilities and additional bioburden reduction during assembly and testing (Chen et al., 2023). Encapsulated, enclosed, or mated bioburdens are challenging to reduce through additional bioburden reduction (Kminek et al., 2019); however, if they are sufficiently sterilized, re-contamination risk is small. In contrast, the exposed surfaces on the spacecraft components could be easily re-contaminated but re-disinfection is also easy with additional treatment.

For the initial overall bioburden reduction of spacecraft components before assembly, the dry heat method must be suitable if the material is heat resistant. Dry heat has the capability to inactivate various microbial species, as shown in Figure 2, and it reproduces results consistently and can penetrate both surface and deep layers within the components. Using Equation (8), the processing times of dry heat for various processing temperatures obtained in this study can be converted to the dry heat treatment processing times for an optimal reference temperature using *F*_P_-values. Reduction curves were estimated for dry heat treatments at 111.7 and 125.0°C as typically described in the COSPAR guidelines (Supplementary Figure S6; European Cooperation for Space Standardization, 2013a; Kminek et al., 2019; NASA, 2022). Tailings in the curves and data variability were observed after four logs order of reduction. Less than four log spores of initial bioburden would be desirable for accurate bioburden reduction using dry heat.

Techniques other than dry heat should be carefully used for non-heat-resistant components in initial and additional bioburden reduction. IPA sterilized *D. radiodurans*, *B. diminuta*, and *A. niger* immediately, whereas H_2_O_2_ (solution and vapor) sterilized *D. radiodurans* and *B. diminuta* immediately. Although IPA treatment is a convenient technique, it was ineffective concerning *B. atrophaeus* (Figure 4A), and H_2_O_2_ was not effective immediately for *B. atrophaeus* and *A. niger* (Figure 4B). Chemical treatments must be more effective when applied with wiping than when used alone for bioburden reduction. UV light is a valuable technique for surface sterilization as *B. atrophaeus* can be sterilized relatively easily (Figure 3). In contrast, when UV light is used to reduce the bioburden, being aware of microorganisms that show higher resistance than *B. atrophaeus*, such as *D. radiodurans* and *A. niger*, is essential. VHP treatment did not sterilize *B. atrophaeus* or *A. niger*, but it achieved two log orders of bioburden reduction in 10 min, even at room temperature (Figure 5). If used with a cleaning process in advance, VHP could be an effective sterilization technique. Plasma treatment produces UV light and heat as secondary factors in addition to the respective sterilization factors (Moisan et al., 2002). Although quantifying each effect is challenging, the bioburden reduction of several microbial species could be achieved (Figure 6), and continuously introducing different types of gases may be helpful for the sterilization. Plasma sterilization can be effective because it can be performed at relatively low temperatures, does not use chemicals, and has no residual properties. In addition to the low-pressure plasma tested in this study, portable atmospheric-pressure plasma may be effective in additional bioburden reduction.

For fitting to the individual systems or situations, the required time for the sterilization can be reduced by optimizing the conditions, such as increasing the voltage for UV light and plasma treatments, increasing the number of lamps and adjusting the distance to the light source for UV treatment, changing the pressure and agent concentration for VHP, changing the agent concentration for H_2_O_2_, and the temperature and humidity for all bioburden reduction techniques, or using a combination of each sterilization technique. For example, VHP batch treatment could sterilize *Geobacillus stearothermophilus* spores to approximately five log orders when treated with heat (Chung et al., 2006), unlike this study that performed treatments at room temperature. Furthermore, less exposure to an aerosolized hypochlorous solution is required to obtain similar sporicidal activity levels at 100%RH than at 70%RH (Ishikawa et al., 2019). Considering that the effectiveness of sterilization treatments varies among different microorganism species, bioburden reduction methods should be evaluated using the most tolerant microorganisms for each technique as model organisms. For instance, *A. niger* should be employed to assess the UV treatment, and *D. radiodurans* should be used for argon plasma treatments. Furthermore, it should be noted that solid surface bioburden may result in less uniform microbial reduction. Hence, sterilization methods should be designed under conditions where the initial bioburden is sufficiently low. For this purpose, it could be valuable to reduce contamination through pre-cleaning before the bioburden reduction process.

**Figure 7:**
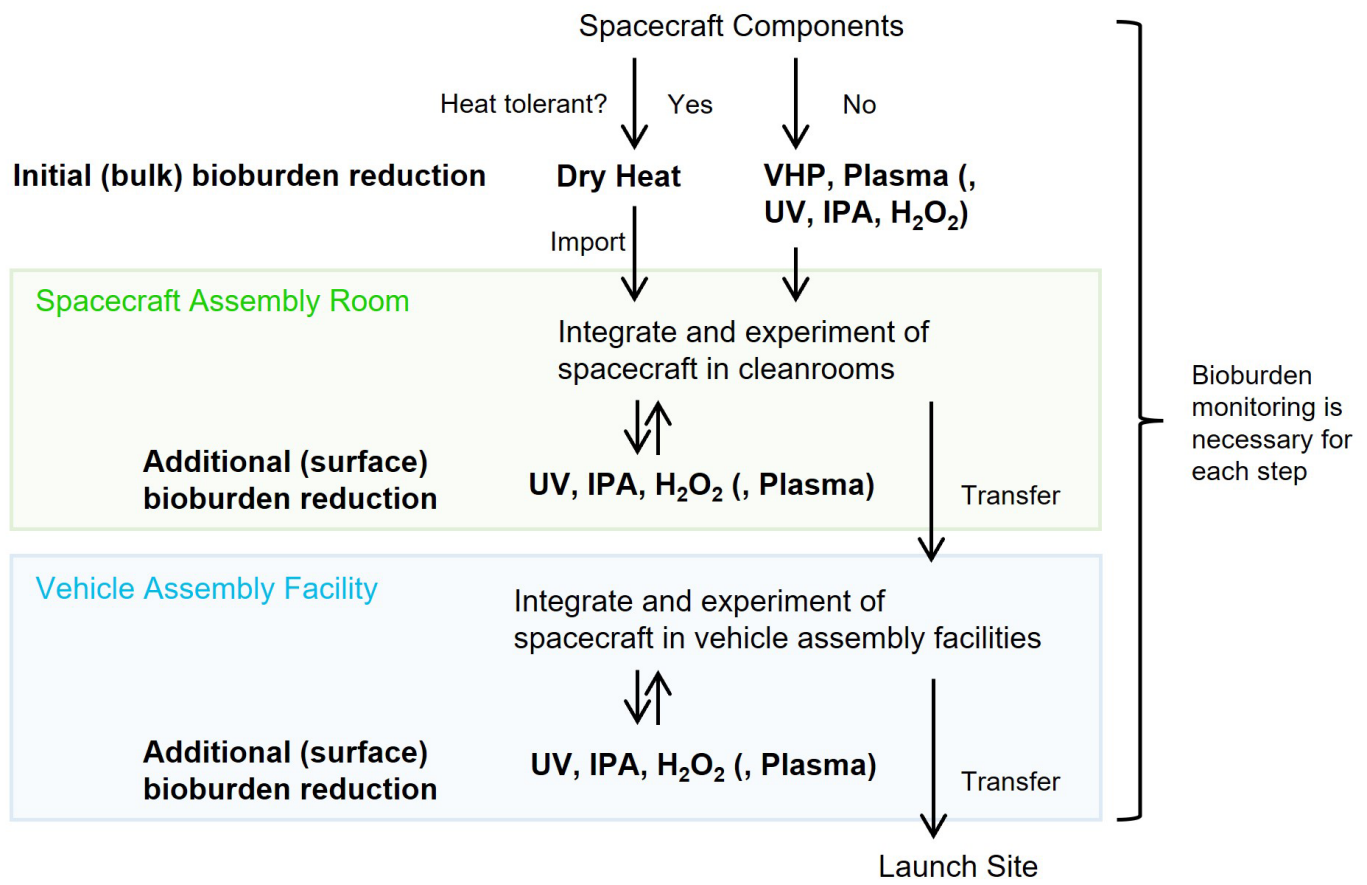
Conceivable bioburden reduction alternatives to integrate into spacecraft.

The anticipated process of the spacecraft components from the introduction into the facility to assembly and launch is shown in Figure 7. Dry heat treatment is appropriate for initial bioburden reduction if the components are heat-resistant. UV light, VHP, and plasma treatment are options for non-heat-resistant components. IPA is the primary method for additional surficial bioburden reduction in assembly cleanrooms. However, H_2_O_2_, UV light, or portable plasma treatment could be alternatives if IPA is insufficient to reduce the bioburden. These sterilization techniques, combined with storing components in a high-efficiency particulate air-filtered space and reducing the bioburden in liquids using autoclaves and filter filtration, would provide a system for launching a sterile spacecraft. Bioburden monitoring at each of these stages is essential. Bioburden reduction confirmation through incubation is the standard method; however, this technique is time-consuming, and detection is limited to microorganisms that form colonies on solid media. Therefore, using a rapid, culture-independent detection method is preferred.

This study quantitatively compared the effectiveness of bioburden reduction techniques on typical resistant microorganisms that can attach to spacecraft (Table 1). The results of this study are useful for selecting the appropriate sterilization methods for planetary protection, and this study will also help planetary protection engineers to choose the most tolerant model organism when they assess each bioburden reduction method. However, other more resistant microorganisms may be present in the natural system. Future studies should expand our knowledge of bioburden reduction methods to improve the techniques, including using different detection methods, expanding the range of microorganisms and developing new sterilization methods. These efforts should aim to reduce the risk of contaminating other planets.

## 5 Conclusion

Systemically comparing the various sterilization techniques under the same experimental conditions revealed the different sensitivities among microorganisms. *Bacillus atrophaeus* showed the highest resistance to dry heat, alcohol, 7.5% H_2_O_2_, and VHP. *Deinococcus radiodurans* and *A. niger* were more resistant to UV than *B. atrophaeus*, and *D. radiodurans* showed the highest resistance to argon plasma treatment. Notably, IPA, widely used for bioburden reduction in planetary protection, had no inactivation effect on *B. atrophaeus* spores. These experimental results provide an accurate estimation of the effectiveness of the sterilization techniques during spacecraft assembly. The method used for bioburden reduction depends on the compatibility of the spacecraft components to withstand the particular technique. Evaluating the damage caused to the spacecraft components by sterilization and selecting and using components that can withstand the entire sterilization process would be effective for future space exploration missions compatible with planetary protection policies and requirements.

## Data availability statement

The raw data supporting the conclusions of this article will be made available by the authors, without undue reservation.

## Author contributions

SK: Conceptualization, Data curation, Funding acquisition, Investigation, Methodology, Validation, Writing – original draft, Writing – review & editing, Formal analysis, Project administration, Resources. SI: Formal analysis, Methodology, Validation, Writing – original draft, Writing – review & editing. NH: Data curation, Methodology, Writing – review & editing. KF: Investigation, Writing – review & editing. YI: Funding acquisition, Investigation, Writing – review & editing, Project administration, Resources. SS: Conceptualization, Funding acquisition, Investigation, Methodology, Supervision, Validation, Writing – original draft, Writing – review & editing, Project administration, Resources.
