# Supplementary material for: Bacterial and fungal bioburden reduction on material surfaces using various sterilization techniques suitable for spacecraft decontamination
Source: Front Microbiol. 2023 Dec 13;14:1253436. doi: 10.3389/fmicb.2023.1253436 (PMC10751312; doi:10.3389/fmicb.2023.1253436)
Supplement: Supplementary file 1 [file Data_Sheet_1.pdf]

Supplementary Material

Bacterial and fungal bioburden reduction on material surfaces using various sterilization techniques suitable for spacecraft decontamination

Shunta Kimura[1, 2, 3*], Shu Ishikawa[4], Nobuya Hayashi[5], Kazuhisa Fujita[1, 6], Yuko Inatomi[1, 2, 3] and Shino Suzuki[1, 2, 3, 7*]

*** Correspondence:** Shunta Kimura: kimura.shunta@jaxa.jp, Shino Suzuki: suzuki.shino2@jaxa.jp

1 Supplementary Figures and Tables

1.1 Supplementary Figures

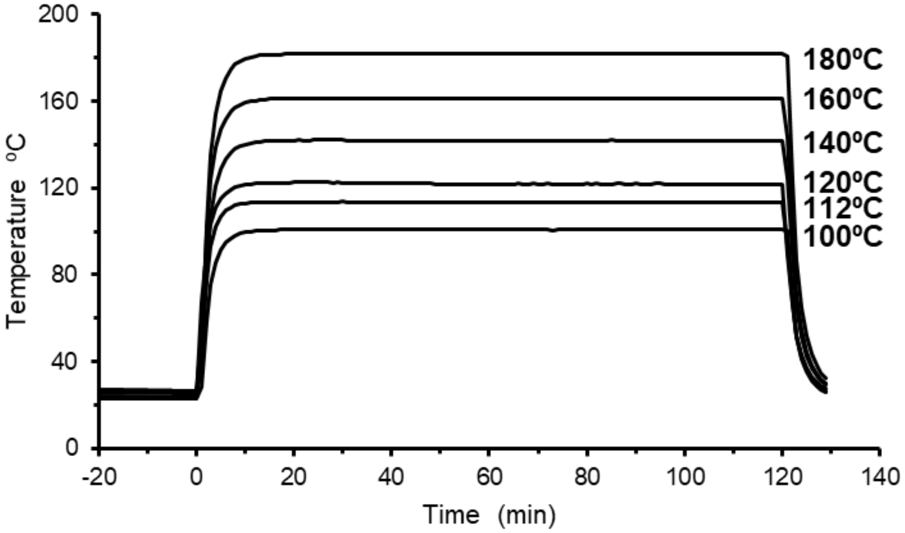

Supplementary Figure S1. Typical temperature profiles on inoculated coupon surfaces during dry heat treatment. Oven temperatures are indicated.

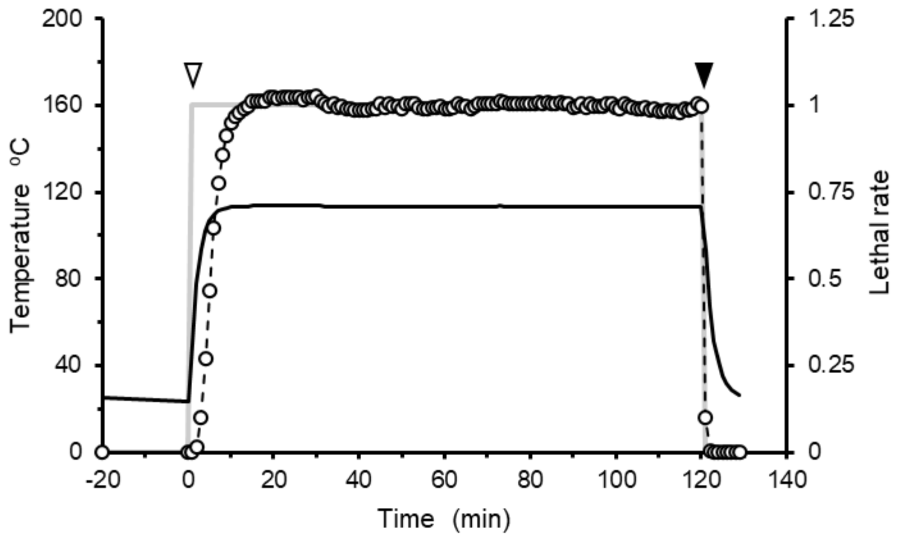

Supplementary Figure S2. The typical temperature profile of dry heated coupon surfaces in a 112ºC-oven (solid line) and lethal rate over time (opened circles with a broken line) is shown. The theoretical lethal rate without a heating and cooling period is also shown as a gray-colored line. The area enclosed by the curve of the lethal rate is the F_P-value at a processing temperature of 113.4ºC. White and black arrowheads indicate the start and end of the dry heat treatment, respectively.

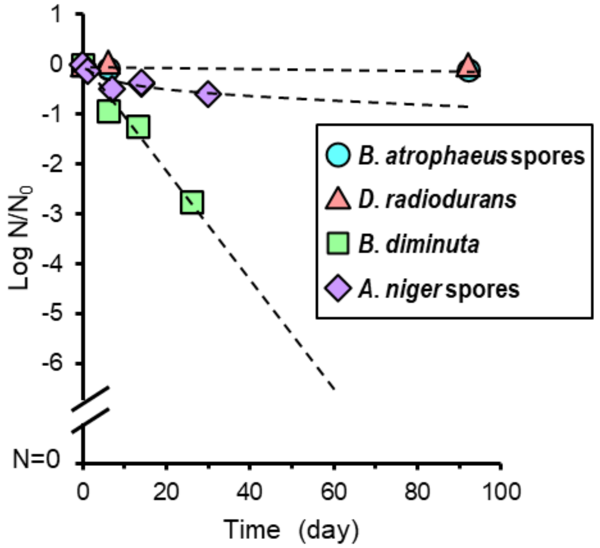

Supplementary Figure S3. Reduction curves during storage in desiccator of dry *B. atrophaeus* spores, *D. radiodurans*, *B. diminuta* and *A. niger* spores.

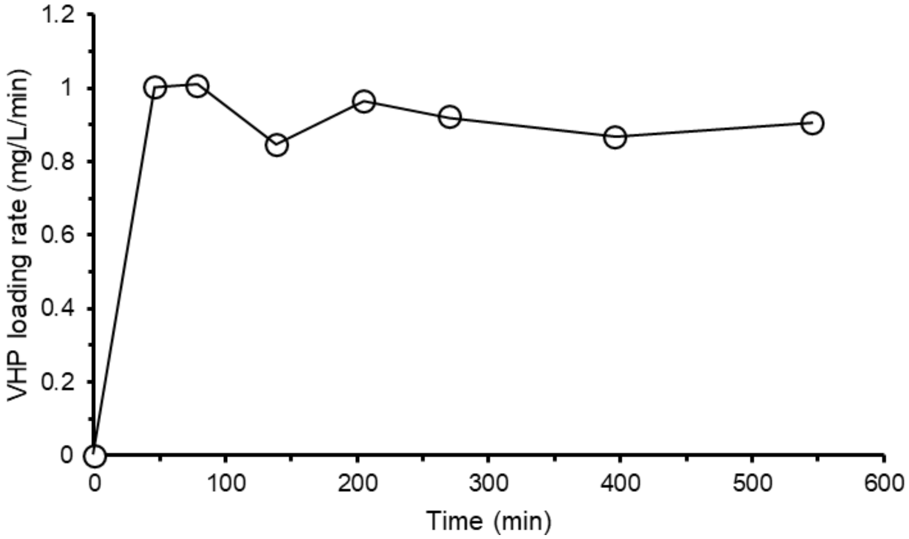

Supplementary Figure S4 VHP loading rate to the vacuum chamber during continuous VHP treatment.

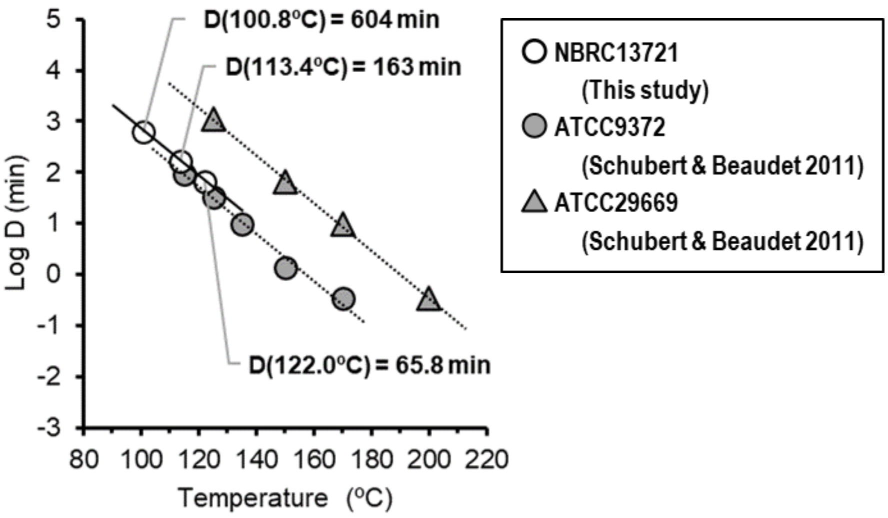

Supplementary Figure S5 The *D*-values logarithm was plotted against temperature to present thermal resistance curves. *D*-values obtained in this study (opened circles) were compared to data of Schubert and Beaudet (2011) (gray-colored circles and triangles). *B. atrophaeus* NBRC13721 and ATCC9372 are synonymous. *Bacillus* sp. ATCC29669 is a heat-resistant strain.

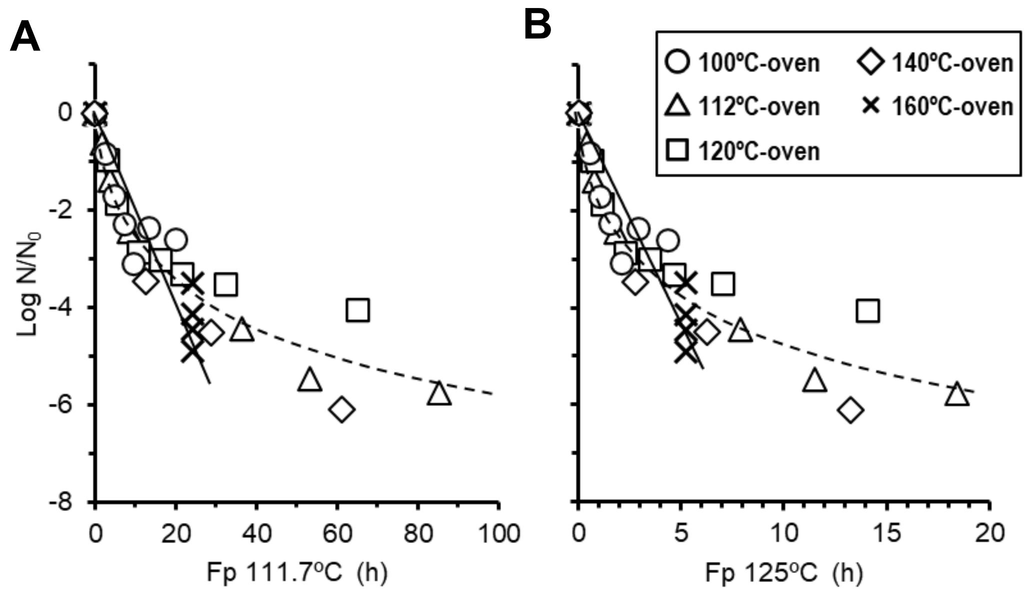

Supplementary Figure S6 Hypothetical survival curves of dry heated *B. atrophaeus* spores with processing time values using a 100-, 112- 120-, 140-, and 160ºC-oven were converted to that of 111.7ºC (**A**) and 125.0ºC (**B**) using the F_P-value as described in Equation (8).

1.2 Supplementary Tables

Table S1. Microorganisms used in this study

**Microorganism**	**Strain**	**Synonymous ATCC number**	**Description**	**Physiological properties in this study**
Bacillus atrophaeus	NBRC 13721	ATCC 9372	A gram-positive bacterium. Resistant to various stresses, including heat, UV, and some antimicrobial agents.	Air-dried spores harvested from the agar plates after 10 days of incubation
Deinococcus radiodurans	NBRC 15346	ATCC 13939	A gram-positive bacterium. Resistant to UV, gamma radiation, and drying.	Air-dried vegetative cells harvested from liquid culture at the mid-log growth phase
Brevundimonas diminuta	NBRC 14213	ATCC 19146	A gram-negative bacterium. Resistant to drying and permeable to filters.	Air-dried vegetative cells harvested from liquid culture at the mid-log growth phase
Aspergillus niger	ATCC 16888	-	A filamentous fungus. Resistant to drying, UV and oxidative stress.	Air-dried spores harvested from the agar plates after 10 days of incubation

Supplementary Table S2. F_P-values of each dry heat treatment.

Heating time (min)	Required F_P-value* (min)	F_P-value (min) †					
		$F_{P\ 100.8°C}$ (100°C-oven)	$F_{P\ 113.4°C}$ (112°C-oven)	$F_{P\ 122.0°C}$ (120°C-oven)	$F_{P\ 141.9°C}$ (140°C-oven)	$F_{P\ 161.3°C}$ (160°C-oven)	$F_{P\ 181.8°C}$ (180°C-oven)
6	≥5.4	1.8	1.9	1.6	1.2	0.8	(0.7)
12	≥10.8	6.9	7.4	6.8	5.2	4.8	(5.0)
20	≥18.0	14.7	15.5	15.2	13.6	12.8	(12.7)
30	≥27.0	24.7	25.5	25.2	23.6	22.8	(22.7)
45	≥40.5	39.7	40.5	40.2	38.6	(37.9)	(37.7)
60	≥54.0	54.7	55.4	55.2	53.6	(52.8)	(52.7)
80	≥72.0	74.7	75.4	75.2	73.6	(72.8)	(72.7)
120	≥110.0	114.7	115.4	115.2	113.6	(112.8)	(112.7)

*) F_P-value used as selection criteria for survival rate data for kinetic analysis in this study.

†) F_P-values were calculated using a *z*-value of 20°C. Numbers in parentheses indicate that *B. atrophaeus* spores were sterilized under that condition.

Supplementary Table S3. The thickness (µm) of microbial spores or cells attached to the coupons. The average height over 100 µm horizontally was measured using a laser microscope ($n \geq 8$).

**Species**	*B. atrophaeus*	*D. radiodurans*	*B. diminuta*	*A. niger*
**Average**	0.9	6.7	4.7	3.9
**Standard deviation (SD)**	0.6	2.4	0.7	1.4
